# Repeat protein scaffolds: ordering photo- and electroactive molecules in solution and solid state†
†Electronic supplementary information (ESI) available: Supplementary Fig. 1–7, experimental details, characterization and NMR data for all new compounds. See DOI: 10.1039/c6sc01306f


**DOI:** 10.1039/c6sc01306f

**Published:** 2016-05-24

**Authors:** Sara H. Mejías, Javier López-Andarias, Tsuneaki Sakurai, Satoru Yoneda, Kevin P. Erazo, Shu Seki, Carmen Atienza, Nazario Martín, Aitziber L. Cortajarena

**Affiliations:** a IMDEA-Nanoscience , Campus de Cantoblanco , E-28049 Madrid , Spain; b CIC biomaGUNE , Paseo de Miramón 182 , E-20009 Donostia-San Sebastian , Spain; c Departamento de Química Orgánica I , Facultad de Ciencias Químicas , Universidad Complutense de Madrid , E-28040 Madrid , Spain . Email: nazmar@ucm.es; d Department of Applied Chemistry , Graduate School of Engineering , Osaka University , Japan; e Ikerbasque , Basque Foundation for Science , Ma Díaz de Haro 3 , E-48013 Bilbao , Spain

## Abstract

The precise control over the organization of photoactive components at the nanoscale is one of the main challenges for the generation of new and sophisticated macroscopically ordered materials with enhanced properties. In this work we present a novel bioinspired approach using protein-based building blocks for the arrangement of photo- and electroactive porphyrin derivatives. We used a designed repeat protein scaffold with demonstrated unique features that allow for the control of their structure, functionality, and assembly. Our designed domains act as exact biomolecular templates to organize porphyrin molecules at the required distance. The hybrid conjugates retain the structure and assembly properties of the protein scaffold and display the spectroscopic features of orderly aggregated porphyrins along the protein structure. Finally, we achieved a solid ordered bio-organic hybrid thin film with anisotropic photoconductivity.

## Abstract 2

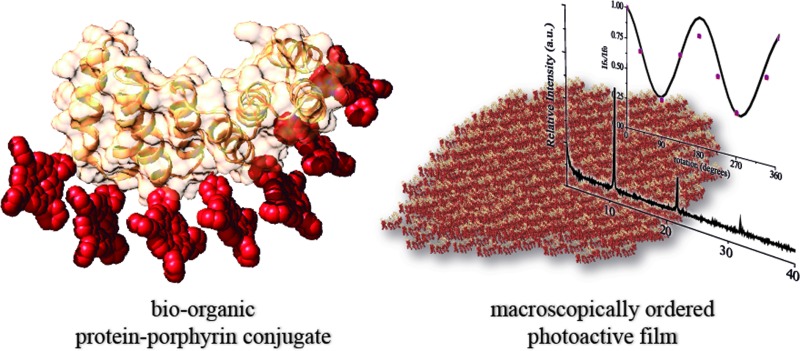
An innovative approach is investigated in which a protein building block is designed to organize porphyrin molecules. The strategy is based on a modular protein unit with tunable properties including stability, function and self-assembly.

## Introduction

One of the main challenges in current science is the construction of highly ordered materials bearing photonic and/or electronic active units.^1^,^2^ Nowadays, many bottom-up approaches have been undertaken to control the organization and morphology of electro- and photoactive components at the nanometer scale, enabling the formation of supramolecular architectures that typically result in a significant improvement of their properties.^3^–^5^ Nonetheless, the majority of these methodologies lack the selective positioning and orientation of the different functional components, and the control at different length scales is still challenging. In addition, poor monodispersity of the aggregates is another drawback. However, templating these components with bioinspired scaffolds can provide the control over the location, monodispersity and chirality needed for technological purposes. Furthermore, bioinspired self-assembling based on biological building blocks including peptides, proteins and DNA has recently been explored to construct sophisticated macroscopic materials that are hierarchically organized through self-assembly.^6^–^14^

Promising examples show the use of biomolecules for ordering organic materials. For example, DNA^15^,^16^ and self-assembling peptide nanotubes^17^ have been used to arrange photo- and electroactive molecules such as fullerenes. Similarly, helical peptides and polymer-peptide have been applied to pattern semiconductors and organic chromophores into ordered structures.^18^,^19^ Porphyrins represent an important class of photo- and electroactive molecules which have been supramolecularly organized using a variety of biomolecules. Thus, as representative examples, the tobacco mosaic virus,^20^ peptides,^21^–^23^ and nucleic acids^24^,^25^ have been used as scaffolds for precisely controlling the position and orientation of porphyrins. These works represent the proof of concept that bioinspired assembly can be successfully used to generate more efficient organo-electronic devices. However, an important limitation of these approaches is how to transfer hierarchically this organization to the macroscopic level in the solid state. The combination of both the generation of bio-organic conjugates with photonic and/or electronic properties and the macroscopic organization of these building blocks could lead to the construction of functional bulk biomaterials with applications in fields such as organic electronics and photovoltaics to name a few. For this end, the design of simple biological building blocks with defined functionalities able to assemble into structured materials is a key issue.

## Results and discussion

In this work, we focus on the design and application of a particular type of biomolecular scaffold based on engineered repeat proteins which encompass the structural simplicity of DNA and short peptides and the functional versatility of proteins.^26^ Specifically, the repeat module of choice is a designed consensus tetratricopeptide repeat protein (CTPR).^27^,^28^ The CTPR module is a 34 amino acid sequence that folds in a helix-turn-helix structure. Few conserved residues define the TPR fold, which permits the rational manipulation of the protein scaffold while retaining the structure. CTPR repeats combined in tandem form superhelical arrays with different number of repeats from 2 to 20, so-called CTPR2 to CTPR20 proteins.^29^,^30^ Previous works on this protein system showed that their stability,^31^,^32^ and ligand binding properties^33^,^34^ can be tuned in a predictable manner, which opens the opportunity to tailor the properties of the individual building block in a modular manner for specific applications.

In line with the creation of protein-based nanostructured materials, we have recently shown that CTPR modules have intrinsic self-assembling properties.^35^–^37^ CTPR proteins retain their structure and functionality in the solid state, creating a nanostructured macroscopic film through head-to-tail and side-to-side interactions similar to those presented in their crystalline form.^36^ This was the first example in which a solid-state protein film displayed both structural and functional properties.

Taking into account these two unique features of the CTPR scaffold: (i) the structural understanding of the CTPR module and the small number of conserved residues that define the CTPR fold to accurately modify selected positions of the amino acid sequence without perturbing its structure and, (ii) the extraordinary capability of CTPR scaffolds to form nanostructured solid films, these proteins offer an unique opportunity to fabricate well-ordered hybrid bio-organic materials. To the best of our knowledge, this combination still remains unexplored.

Hence, we have designed a modified CTPR protein as scaffold for assembling a specific number of porphyrin chromophores in a defined distance and orientation. Based on the crystal structure of the CTPR protein,^34^ different amino acid positions were selected to arrange the chromophores along the CTPR superhelix that allow for efficient π–π interactions between the molecules. The selected positions are not conserved between CTPR modules, thus it is expected that their modification will not affect the structure of the protein template.

**Fig. 1:**
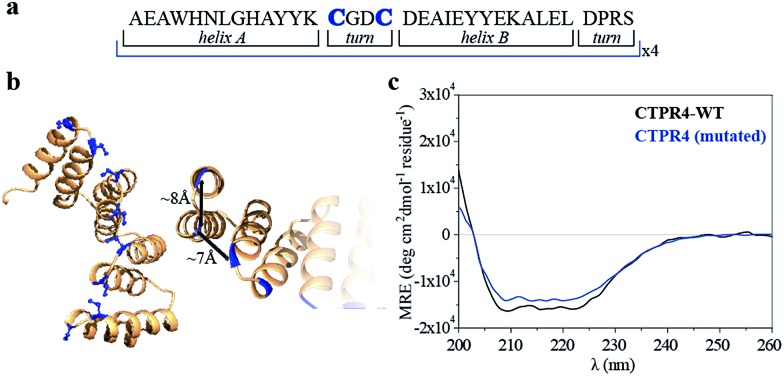
(a) Sequence of amino acids of the repeat module, highlighting the positions 14 and 17 that have been mutated to cysteine. (b) Ribbon representation of mutated **CTPR4** protein structural model based on the structure of **CTPR8** (PDB ID:; 2HYZ). The mutated cysteine residues for the porphyrin attachment are highlighted in blue. (c) CD spectra of mutated **CTPR4** comparing with the original **CTPR4**.

Specifically, two cysteine residues were introduced in each repeated unit to provide unique reactivity for the immobilization of the porphyrin derivatives. A CTPR protein with four identical repeats was generated resulting in a protein with eight quasi-equally spaced cysteine residues in four loops of the protein (Fig. 1a and b). The distance between the Cys side chains is between 7 and 8 Å, in the order of the distance required to establish π–π interactions between the porphyrin rings. As expected, the mutations did not significantly affect the helical structure of the protein scaffold (Fig. 1c).

**Fig. 2:**
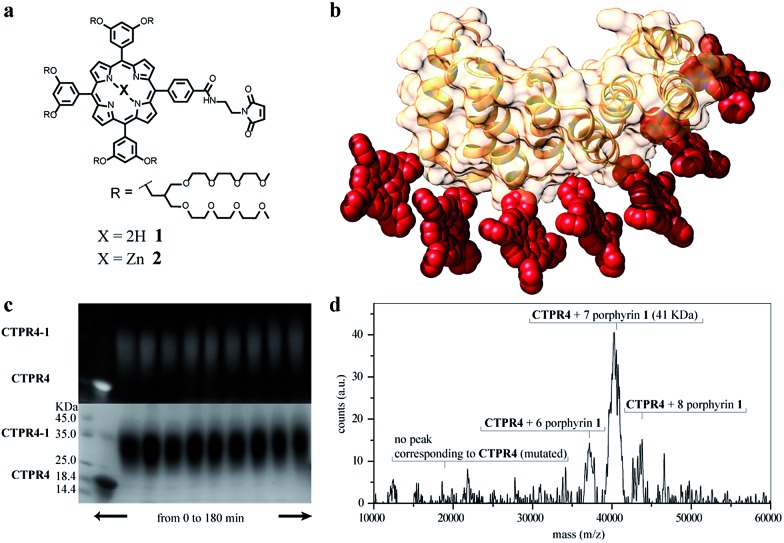
(a) Molecular structure of porphyrin derivatives **1** and **2**. (b) 3D model structure of the **CTPR4-1** conjugate (triethylene glycol chains are omitted for clarity). (c) SDS-PAGE gel electrophoresis of the **CTPR4-1** conjugation at different reaction times (20–180 every 20 min, lanes 3–11). Molecular weight marker (lane 1) and **CTPR4** control with fluorescent loading buffer (lane 2). The gel is imaged using UV-light to monitor the fluorescence of the porphyrins (upper panel) and after Coomassie Blue staining (lower panel). (d) MALDI-TOF spectrum of the final conjugation reaction in **CTPR4-1**. A main peak appears at 41 kDa that corresponds to the mass of **CTPR4** protein (19 163 Da) with 7 porphyrins (MW of **1**: 3160 Da).

Besides, in the design of the porphyrin moiety two important requirements should be considered: (i) porphyrin molecules must exhibit high solubility in water but avoiding charged functional groups, since undesired interactions with charged side-chains of the amino acids in the protein should be eliminated and, (ii) an efficient cross-linker must be placed in the porphyrin structure in order to promote quantitative or quasi-quantitative conjugation with the eight cysteine residues of the designed protein. With this in mind, two porphyrin derivatives were synthesized, free-base and zinc-metalloporphyrin (**1** and **2**, respectively), decorated with twelve triethylene glycol water-soluble tails and a maleimide reactive group as efficient cross-linker for the conjugation reaction (Fig. 2a and see ESI Scheme S1†). For further information about the design, synthesis and characterization of porphyrin derivatives, see ESI.† 38,39

The designed protein (**CTPR4**) and porphyrin moieties (**1** and **2**) (Fig. 2a and b) were conjugated using the maleimide–cysteine chemistry. At different times during the conjugation reaction, the analysis of the samples by gel electrophoresis showed that a conjugate of higher molecular weight than **CTPR4** protein (19 163 Da, see ESI Fig. S1†) and porphyrins (MW_1_ = 3160 Da, MW_2_ = 3223 Da) was obtained even at the shortest reaction time (Fig. 2c and see ESI Fig. S2†). When the gel was imaged without staining, the fluorescence signal of the porphyrin could be detected showing a new band between 31 and 45 kDa, corresponding to the molecular weight expected for the **CTPR4-1** or **CTPR4-2** conjugates (Fig. 2c – top). Moreover, the staining of the protein with Coomassie Blue confirmed that the higher molecular weight band was composed of both protein and porphyrin (Fig. 2c – bottom).

To quantify the number of porphyrin molecules bound per protein in the conjugation reaction, mass spectrometry was used (Fig. 2d and see ESI Fig. S2†). After 4 h reaction time, the peak corresponding to the **CTPR4** protein disappeared and a most abundant peak at 41 kDa appeared, which corresponds to a **CTPR4** protein covalently linked to seven porphyrin units, together with two smaller peaks both at higher and lower molecular weights, corresponding to the conjugate with eight and six porphyrins, respectively. The purification of the protein–porphyrin conjugates from the excess of free porphyrin is an essential step for further use and characterization of homogeneous hybrid structures. **CTPR4-1** and **CTPR4-2** purification was successfully carried out using size exclusion chromatography (see ESI Fig. S3†). All the characterization experiments were performed using the purified conjugates.

**Fig. 3:**
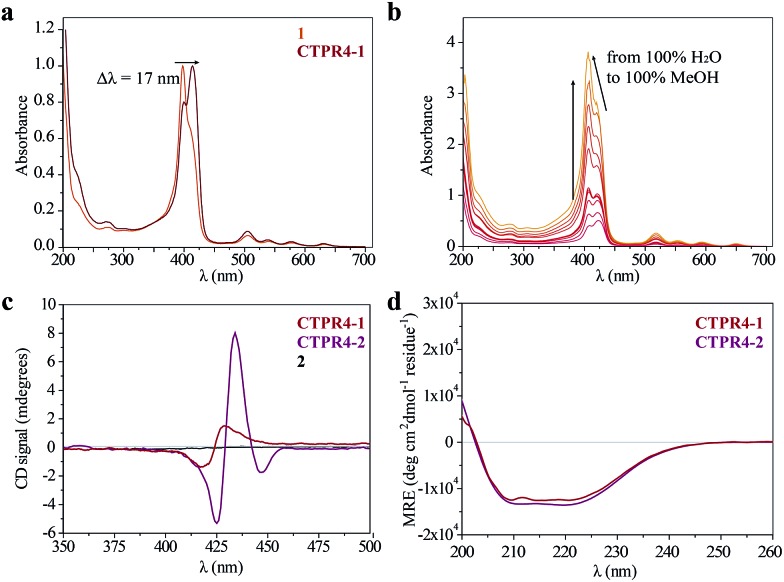
(a) Normalized UV-vis spectrum of **CTPR4-1** conjugate compared to normalized UV-vis spectrum of **1** in PBS buffer. (b) UV-vis spectra of **CTPR4-1** (0.56 μM) at different methanol concentrations (from 0 to 100%). (c) CD spectra of porphyrin **2** and **CTPR4-1** and **CTPR4-2** conjugates (1.70 μM) in PBS buffer in the spectral region of the porphyrin absorption. (d) CD spectra of **CTPR4-1** and **CTPR4-2** conjugates in PBS buffer in the spectral region of the protein's secondary structure absorption.

As has previously been mentioned, the distinctive properties of the CTPR scaffold allowed for the design of a **CTPR4** protein with an appropriate distance between the reactive moieties that, considering the rotational freedom of both the protein side chains and the linked porphyrins, will enable an efficient π–π intermolecular interaction between the porphyrin rings. It is well established that porphyrins have the ability to self-aggregate noncovalently to form H-aggregates (face-to-face) or J-aggregates (side-to-side), these states are characterized by a shift on the Soret absorption band towards the blue or red, respectively, compared to the monomeric absorption band.^40^–^45^ Thus, the UV-vis spectra of both porphyrins as references and **CTPR4-1** and **CTPR4-2** conjugates were recorded in order to obtain more information about the conformation of the porphyrins in the conjugates. The absorption spectrum of porphyrin **1** in a PBS buffer solution presents an intense Soret band at 406 nm and four Q-bands in the region between 500 and 700 nm. Meanwhile, porphyrin **2** presents the Soret band at 425 nm and two Q-bands at 555 and 595 nm. In contrast, the UV-vis spectra for **CTPR4-2** and **CTPR4-1** conjugates showed a red shift of 5 and 17 nm in the Soret band of the porphyrin, respectively, compared with the corresponding free porphyrins (Fig. 3a and see ESI Fig. S4†). These shifts suggest the formation of a J-type aggregate of porphyrins in both conjugates, certainly induced by the geometry of the protein and the position of the cysteine residues in the framework. To further confirm the π–π interaction between porphyrin moieties within the conjugates, UV-vis spectra were measured at different H_2_O : MeOH ratio leading to an increased intensity and a blue-shift of the Soret band while the percentage of methanol increases (Fig. 3b and see ESI Fig. S4†). This fact is in agreement with the disruption of the π–π interaction when protein denaturation is achieved with an organic polar solvent.

Circular dichroism (CD) studies in solution were performed on the **CTPR4-1** and **CTPR4-2** conjugates. In the first case, the **CTPR4-1** conjugate showed a bisignate signal with a negative Cotton effect at 417 nm, a positive Cotton effect at 428 nm and a zero cross point at 423 nm (Fig. 3c). The presence of these Cotton effects, that is, exciton-coupled circular dichroism, is indicative of a close proximity between the chromophores, as previously reported.^46^–^48^ In the second case, a strong trisignate signal with a negative Cotton effect at 425 nm, positive Cotton effect at 434 nm and another negative Cotton effect at 446 nm with zero cross points at 429 and 442 nm was shown for the **CTPR4-2** conjugate (Fig. 3c). Multisignates in the Soret band have previously been ascribed in the literature to strong π–π stacking interactions between close porphyrins.^25^,^49^ Thus, these dichroic signals in the porphyrin absorption region demonstrated the transfer of chirality from the CTPR protein scaffold to the porphyrin molecules, since free porphyrins **1** and **2** lacked optical activity in their CD spectra (Fig. 3c). Furthermore, the feature CD signal for alpha-helical secondary structure of the **CTPR4** protein was totally retained in both **CTPR4-1** and **CTPR4-2** conjugates in PBS buffer (Fig. 3d). This result confirmed the successful ability of CTPR proteins to be used as a robust scaffold for ordering organic chromophores, preserving its secondary and tertiary structure even when six to eight porphyrin molecules per protein were incorporated. It is important to note that the final molecular weight of both conjugates represent more than twice the initial one corresponding to the protein alone; however, the structural integrity of the biological framework is remarkably conserved. Moreover, a denaturalization was also carried out for both conjugates through CD measurements. No appreciable dichroic signal in the range of Soret band of the porphyrins was observed after adding methanol, which confirms the rupture of π–π interactions when the protein scaffold is unstructured (See ESI Fig. S5†).

**Fig. 4:**
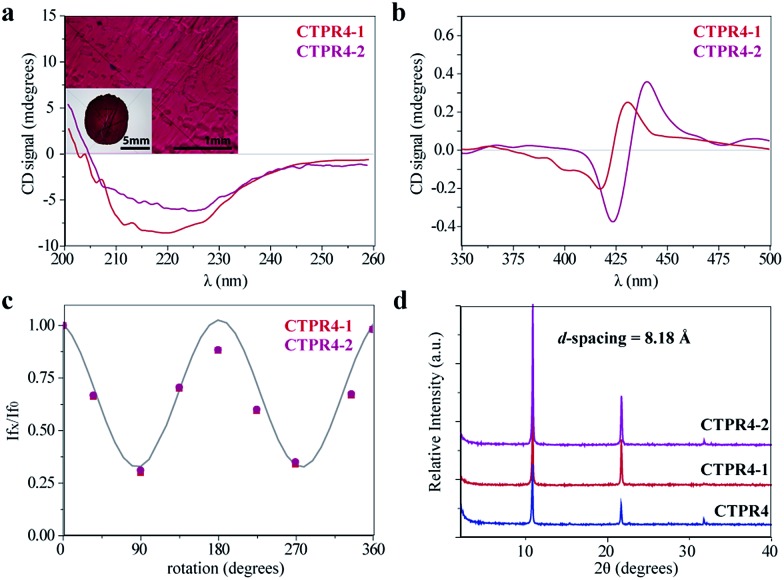
(a) CD spectra of the **CTPR4-1** and **CTPR4-2** conjugates in solid thin films in the spectral region of the protein's secondary structure absorption. Inset: photographs of the **CTPR4-2** film at different magnifications (scale bars: 5 and 1 mm). (b) CD spectra of the **CTPR4-1** and **CTPR4-2** conjugates in solid thin films in the spectral region of the porphyrin absorption. (c) Fluorescence anisotropy of the **CTPR4-1** and **CTPR4-2** films. (d) XRD diffractogram of **CTPR4**, **CTPR4-1** and **CTPR4-2** thin films.

The intrinsic capability of CTPR proteins to assemble into highly ordered thin films makes them good candidates to organize porphyrin arrays in the solid state.^36^ However, the formation of protein-based solid ordered materials has been achieved in only a very few protein systems. Furthermore, the combination with the building-block tunability is unique of the CTPR scaffold. In this sense, ordered thin films with both conjugates were effectively generated under similar experimental conditions to those described for CTPR proteins alone. Importantly, the structural integrity of the protein block and the porphyrin arrangement was tested by CD experiments (Fig. 4a and b). Fig. 4a and b show how the secondary structure of **CTPR4** scaffold remained alpha helical with no evidence of any significant structure other than alpha helix, and how the chiral environment of the porphyrins was maintained in both conjugates. Moreover, as has previously been reported, CTPR proteins are macroscopically aligned in the solid film form,^36^,^37^ thus it was expected that our conjugates would also be aligned. To shed light on this fact, fluorescence anisotropy measurements and X-ray powder diffraction (XRD) were carried out. On the one hand, Fig. 4c shows the change in the fluorescence intensity of the porphyrin moieties when the emission polarizer was rotated from 0 to 360°. The signal showed clear maximum and minimum values, which indicates the anisotropy of the sample. The data can be well fitted to a sine wave function with maximum to minimum peak distance of 107°, in agreement with the alignment phase determined for the CTPR films. This result indicates that the macroscopic ordered pattern of the film is indeed imposed on the porphyrins that are, otherwise, isotropic.

On the other hand, a deep understanding on the organization of the films was reached by XRD experiments. The XRD pattern of a film obtained from **CTPR4** protein showed a set of three clear and intense sharp peaks at 2*θ* = 10.82, 21.64 and 32.05° that could correspond to a lamellar packing with a periodical *d*-spacing of 8.18 Å (Fig. 4d). A similar *d*-spacing has been previously observed in other films based on repeat proteins and α-helical coiled-coil proteins, being related to the meridional spacing for an α-helix structure, dependent on the angle of inclination.^14^,^37^,^50^ Overall, these data demonstrate the directional orientation of the protein on a surface when a film is formed. Moreover, it is remarkable that the diffraction patterns observed for the films based on the conjugates **CTPR4-1** and **CTPR4-2** present exactly the same reflections peaks as **CTPR4**. These experimental findings corroborate that the long-range order of the protein is preserved even with these number of porphyrins introduced in its structure. This structural feature highlights the robustness of this biological scaffold to be used as an efficient template for ordering organic chromophores not only in solution but in the technologically relevant solid state.

**Fig. 5:**
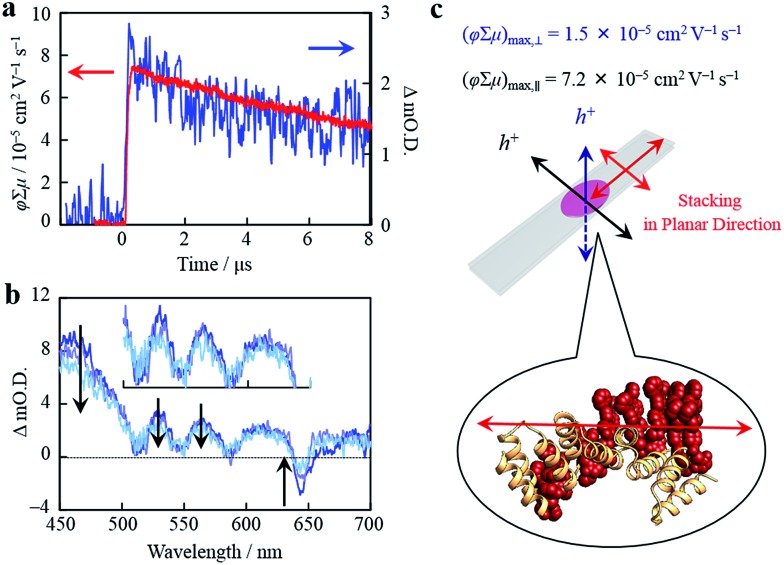
(a) Kinetic traces of transient absorption spectra at 530 nm (blue) and kinetic traces of FP-TRMC (red) of a film of **CTPR4-1**. (b) Snapshot of transient absorption spectra of a film of **CTPR4-1** at *ca.* 0.2 (blue), 3 (light purple), and 8 (turquoise) μs after an excitation. (c) Schematic illustration of a **CTPR4-1** film on quartz and stacking direction of porphyrin arrays together with observed values of conductivity maxima in perpendicular and planar direction to the substrate surface.

Organized porphyrin arrays on the protein scaffold are interesting as charge carrier transporting and photoconductive motifs. The photoconductivity along the arrays in **CTPR4-1** and **CTPR4-2** conjugate films was examined by using the flash-photolysis time-resolved microwave conductivity (FP-TRMC) technique.^51^,^52^ In this non-contact method, charge carriers are photo-generated upon exposure to 355 nm laser pulses to the films. Then, local-scale motions of the generated charge carriers can be probed by monitoring the reflected microwave (∼9.1 GHz) power from the film sample set at the microwave cavity. Upon laser flash, a drop-cast film of **CTPR4-1** showed a conductivity transient with prompt rise and slow decay features (Fig. 5a). The conductivity (*φ*∑*μ*) indicates the product of charge carrier generation efficiency (*φ*) upon photoexcitation and sum of charge carrier mobilities (∑*μ* = *μ*_h_ + *μ*_e_). Furthermore, the same film yielded a clear transient absorption spectra (TAS), where photo-bleach of the neutral and generation of radical cations were observed at the Q-band region of the free-base porphyrins (Fig. 5b).^53^ In fact, the normalized profiles of FP-TRMC and TAS at 530 nm gave almost identical kinetic traces (Fig. 5a), indicating that hole transport is the dominant factor for the observed local-scale photoconductivity of **CTPR4-1** under air. By using a typical absorption coefficient of the radical cation of tetraphenyl free-base porphyrin,^21^*φ* was calculated to be 5.0 × 10^–2^, followed by the evaluation of one-dimensional mobility (*μ*_h_) of 1.5 × 10^–3^ cm^2^ V^–1^ s^–1^. Then, through the same measurement processes, *μ*_h_ of **CTPR4-2** was determined as 1.3 × 10^–3^ cm^2^ V^–1^ s^–1^ (See ESI Fig. S6†). As a control experiment, a non-conjugated protein-based film, that is, a **CTPR4** film resulted in charge carriers by irreversible photo-damage upon laser exposure (see ESI Fig. S7†). Thus, the higher conductivity values for **CTPR4-1** and **CTPR4-2** were due to the local motion of positive charge carriers as a consequence of the porphyrin arrays. Of further interest, we revealed that structural alignment of the arrays in a macroscopic scale gave rise to the anisotropic electrical conductivity. For example, by changing the direction of the **CTPR4-1** sample in the cavity,^54^ (*φ*∑*μ*)_max_ along the perpendicular and parallel directions to the substrate surface were evaluated as 1.5 × 10^–5^ and 7.2 × 10^–5^ cm^2^ V^–1^ s^–1^, respectively (Fig. 5c). This increment of the electrical conductivity along the parallel direction is in agreement with the anisotropy of the film observed by fluorescence measurements (Fig. 4c).

## Conclusions

This work describes an innovative approach in which a protein building block is designed to organize porphyrin molecules. In contrast to recent works in which existing biomolecular structures have been used to arrange similar molecules, here we have developed a strategy based on a modular protein unit with tunable properties including stability, function and self-assembly. The scaffold is based on a simple unit, which allows for a controlled engineering and introduction of reactive functionalities at defined positions for the conjugation of organic molecules. By combination of simple units we can generate longer proteins while preserving the structural understanding. Therefore, we can pattern the reactive moieties on the 3D structure of the scaffold. Here, we evidence this potential by precisely organizing porphyrin molecules on the CTPR scaffold at the distance required to achieve efficient π–π interactions between the rings. In the final conjugates the protein retains its signature helical structure and imposes order and chirality into the porphyrin molecules that show efficient stacking interactions. These results show the potential of this versatile scaffold that could be applied for control grafting of a variety of functional molecules and nanostructural elements.

Another feature that makes the applied system superior to other protein templates is its assembly properties. Prior works on the CTPR scaffold have shown how ordered nanostructures and nanostructured materials can be fabricated from these simple repeated units. Remarkably, in the ordered assemblies the proteins maintain their structure and function. These observations pave the way to their application for the fabrication of solid functional devices. In this work, as a proof of concept, we have shown the formation of ordered films using hybrid protein–porphyrin conjugates. The films obtained displayed the described nanostructured directional order both in the protein and in the photoactive components. Finally, the photoconductivity of the hybrid thin films showed a remarkable anisotropy in agreement with the directional order of the photoactive molecules. The developed approach is simple and should be easily translatable to other systems that require precise order at different length scales to achieve materials and devices with enhanced properties.

## Supplementary Material

Supplementary information
