# Supplementary material for: Repeat protein scaffolds: ordering photo- and electroactive molecules in solution and solid state
Source: Chem Sci. 2016 May 24;7(8):4842–7. doi: 10.1039/c6sc01306f (PMC5905405; doi:10.1039/c6sc01306f)

Electronic Supplementary Material (ESI) for Chemical Science.
This journal is © The Royal Society of Chemistry 2016

Repeat protein scaffolds: ordering photo and electroactive molecules in solution and solid state

Sara H. Mejías, [a],‡ *Javier López-Andarias,* [c],‡ *Tsuneaki Sakurai,* [d] *Satoru Yoneda,*[d] *Kevin P. Erazo,* [a] *Shu Seki,* [d] *Carmen Atienza,** [c] *Nazario Martín**[a, c] *and Aitziber L. Cortajarena**[a, b]

Contents

A. Materials and techniques and experimental procedure *S3-S8*

B. Supplementary Figures *S9-S17*

- MALDI-TOF spectrum of the **CTPR4** mutated with eight cysteine residues S9
- SDS-Page electrophoresis gel of the **CTPR4-2** conjugate and MALDI-TOF spectra of the conjugation reaction in **CTPR4-1**. S10
- Size exclusion chromatogram of the purification process of the conjugate. S11
- UV-*vis* spectrum of **CTPR4-2** and **2** in PBS buffer and UV-*vis* spectra of **CTPR4-2** at different methanol concentrations. S12
- CD spectra of **CTPR4-1** and **CTPR4-2** in methanol. S13
- FP-TRMC kinetic traces of a film of **CTPR4-2** at different time scale. S14
- FP-TRMC kinetic traces of a film of **CTPR4** S15
- ^{1}H NMR and ^{13}C NMR spectra for all new organic compounds. S16-S18

A. Materials and techniques and experimental procedure.

Protein design and purification. Based on a consensus CTPR4 protein, two cysteine residues were introduced in each CTPR repeat at positions 15 and 17 within the loop connecting the helix A and B within the repeat. The mutations were introduced in the gene encoding 1 repeat (CTPR1) by quick-change site directed mutagenesis. The CTPR4 gene was generated from the CTPR1 mutated gene by sequential additions of identical mutated repeats and cloned into pPro-EXHTa vector. The protein was expressed as His-tagged fusion and purified using standard affinity chromatography methods based on previously published protocols[25] for His-tagged CTPR proteins using 0.5% deoxycholic acid in the lysis buffer. The protein was dialyzed into PBS buffer (150 mM NaCl, 50 mM phosphate buffer pH 7.4) and stored frozen at –20°C. The protein concentration was determined by *UV*-absorbance at 280nm using the extinction coefficient calculated from the amino acid composition (51800 M^{-1} cm^{-1}).

Preparation of porphyrin derivatives 1 and 2.

Reagents for synthesis were mostly purchased from Sigma-Aldrich and Acros. Column chromatography was carried out on silica gel 60 (Fluka, 40-63 μm) and preparative thin layer chromatography (PTLC) was performed on silica gel 60 (Fluka, 0.2 mm). IR spectra were recorded on a Bruker Tensor 27 spectrometer equipped with ATR and reported as wavenumbers in cm^{-1} with band intensities indicated as s (strong), m (medium), w (weak), br (broad). ^{1}H and ^{13}C NMR spectra were recorded either on a Bruker DPV 300MHz, a Bruker AV 500MHz or a Bruker AVIII 700MHz and reported as chemical shifts (δ) in ppm relative to tetramethylsilane (δ = 0) at room temperature. Spin multiplicities are reported as a singlet (s), broad singlet (br s), doublet (d), triplet (t) and quartet (q) with proton-proton coupling constants (*J*) given in Hz, or multiplet (m). Matrix-assisted laser desorption ionization (MALDI) mass spectrometry (MS) was performed on a Bruker Ultraflex spectrometer using ditranol or DCTB as matrix.

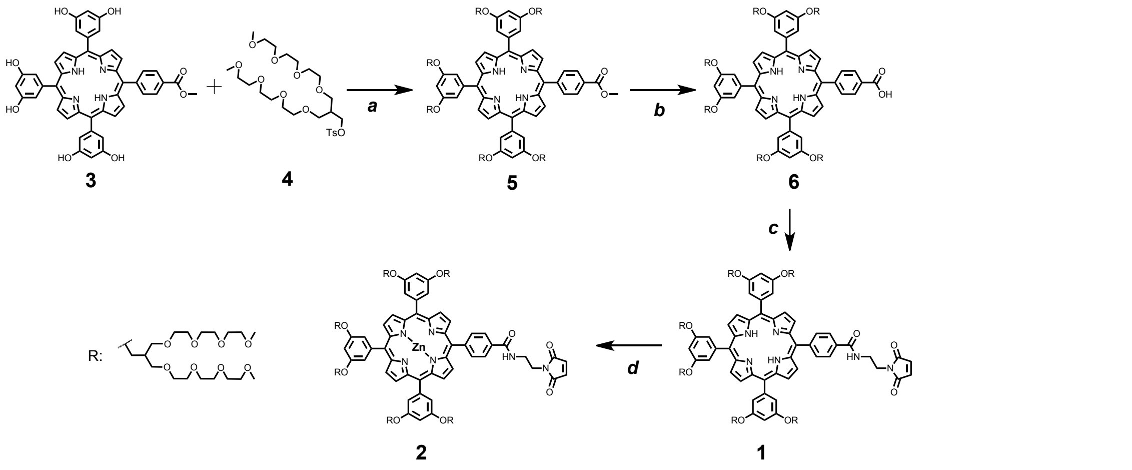

Scheme S1. Synthesis of porphyrin 1 and 2. (a) K_2CO_3, 18-crown-6, DMF, 80 ºC, 64%. (b) KOH, THF/H_2O, reflux, 51%. (c) 2-Maleimidoethylamine trifluoroacetate salt, HBTU, Et_3N, CH_2Cl_2, rt, 73%. (d) $Zn(OAc)_2 \cdot 2H_2O$, CH_2Cl_2:MeOH, rt, 83%.

Compound 5. To a solution of **3** (40 mg, 0.052 mmol) in 5 mL of dimethylformamide, 18-crown-6 (14 mg, 0.052 mmol) and K_2CO_3 (72 mg, 0.52 mmol) were added and the mixture was stirred under Argon and reflux for one hour. To the resulting dispersion, **4** (200 mg, 0.37 mmol) was added and the reaction mixture was allowed to react at 80ºC. After 20 hours, the reaction was cooled down, diluted with CH_2Cl_2 (25 mL) and filtered through celite. The solvent was removed under reduced pressure and the crude product was purified by flash column chromatography (SiO_2, CH_2Cl_2/MeOH 30:1), affording **5** as a reddish sticky oil (102 mg, 64%). IR (ATR): 3318 (w), 2869 (s), 1721 (m), 1589 (m), 1454 (m), 1434 (m), 1352 (m), 1279 (m), 1102 (s), 851 (m), 804 (m); ^{1}H NMR (500 MHz, $CDCl_3$): 8.95 (br s, 6H), 8.78 (d, ^3J = 4.6 Hz, 2H), 8.44 (d, ^3J = 8.0 Hz, 2H), 8.32 (d, ^3J = 8.0 Hz, 2H), 7.37 (d, ^3J = 2.1 Hz, 6H), 6.91 (d, ^3J = 2.1 Hz, 3H), 4.25 – 4.15 (m, 12H), 4.14 (s, 3H), 3.70 – 3.64 (m, 24H), 3.63 – 3.55 (m, 72H), 3.55 – 3.45 (m, 48H), 3.43 – 3.38 (m, 24H), 3.33 – 3.23 (m, 36H), 2.55 – 2.47 (m, 6H), -2.84 (br s, 2H).^{13}C NMR (125 MHz, $CDCl_3$): 167.7, 158.7, 144.0, 134.9, 131.4, 129.8, 128.3, 120.4, 118.8, 114.9, 101.4, 72.2, 71.0, 70.9, 70.8, 70.7, 69.8, 67.0, 59.3, 52.8, 40.5. HR-MS (MALDI, dithranol): 3050.6696 ($[M+H]^+$); calculated for $C_{154}H_{249}N_4O_{56}$: 3050.6760.

Compound 6. Compound **5** (90 mg, 0.029 mmol) was dissolved in 10 mL of a 0.15 M solution of KOH (50 eq) in THF:H_2O (9:1, v:v) and the reaction mixture was heated to reflux and allowed to react overnight. The next day, THF was removed under reduced pressure and around 20 mL of CH_2Cl_2 was added and the solution was transfered to an extraction funnel. A 0.1 M aqueous solution of HCl was added up to neutral pH. The product was extracted in CH_2Cl_2 (3 x 20 mL), dried over Na_2SO_4 and the solvent was removed under reduced pressure, affording **6** as a reddish sticky oil (45 mg, 51%). IR

(ATR): 2871 (s), 1720 (w), 1590 (m), 1455 (m), 1435 (m), 1352 (m), 1106 (s), 851 (m), 804 (m); ^{1}H NMR (700 MHz, $CDCl_3$): 8.95 (br s, 6H), 8.78 (d, ^3J = 4.8 Hz, 2H), 8.54 (d, ^3J = 8.2 Hz, 2H), 8.37 (d, ^3J = 8.2 Hz, 2H), 7.42 – 7.35 (m, 6H), 6.93 – 6.90 (d, ^3J = 2.1 Hz, 3H), 4.26 – 4.16 (m, 12H), 3.72 – 3.64 (m, 24H), 3.64 – 3.57 (m, 72H), 3.56 – 3.47 (m, 48H), 3.45 – 3.39 (m, 24H), 3.33 – 3.23 (m, 36H), 2.54 – 2.49 (m, 6H), -2.85 (br s, 2H).^{13}C NMR (175 MHz, $CDCl_3$): 169.4, 158.3, 147.7, 143.7, 134.6, 128.5, 120.2, 118.5, 114.5, 101.0, 71.8, 70.6, 70.53, 70.4, 69.5, 69.4, 69.4, 59.0, 40.0. HR-MS (MALDI, dithranol): 3036.6469 ([M+H]$^+$); calculated for $C_{153}H_{247}N_4O_{56}$: 3036.6603.

Compound 1. To a solution of **6** (45 mg, 0.0148 mmol), 2-maleimidoethylamine trifluoroacetate salt (5.6 mg, 0.0222 mmol) and HBTU (6.2 mg, 0.0163 mmol) in 1 mL of dry CH_2Cl_2, Et_3N (4.1 µL, 0.030 mmol) was added. The reaction mixture was allowed to react at room temperature. After 15 min, around 10 mL of CH_2Cl_2 was added and the product was cleaned with a saturated solution of $NaHCO_3$ (3 x 5 mL). The organic layer was dried over Na_2SO_4 and the solvent was removed under reduced pressure. The crude product was purified by PTLC (SiO_2, CH_2Cl_2/MeOH 25:1), affording **1** as a reddish sticky oil (34 mg, 73%). IR (ATR): 3320 (w), 2922 (s), 2856 (s), 1712 (m), 1660 (m), 1456 (m), 1352 (m), 1105 (s), 843 (s); ^{1}H NMR (500 MHz, $CDCl_3$): 9.01 – 8.90 (m, 6H), 8.78 (d, ^3J = 4.6 Hz, 2H), 8.29 (d, ^3J = 8.0 Hz, 2H), 8.18 (d, ^3J = 8.0 Hz, 2H), 7.37 (d, ^3J = 1.8 Hz, 6H), 7.24 – 7.17 (m, 1H), 6.91 (d, ^3J = 1.8 Hz, 3H), 6.86 (s, 2H), 4.27 – 4.12 (m, 12H), 4.03 – 3.97 (m, 2H), 3.89 – 3.85 (m, 2H), 3.70 – 3.64 (m, 24H), 3.63 – 3.55 (m, 72H), 3.55 – 3.45 (m, 48H), 3.44 – 3.37 (m, 24H), 3.33 – 3.21 (m, 36H), 2.49 (m, 6H), -2.85 (br s, 2H). ^{13}C NMR (125 MHz, $CDCl_3$): 171.6, 168.3, 158.7, 146.0, 144.1, 135.0, 134.9, 134.0, 125.8, 120.5, 119.0, 114.9, 101.5, 72.3, 72.2, 71.3, 71.0, 70.9, 70.8, 70.3, 69.9, 67.0, 63.8, 59.3, 40.4, 38.1. HR-MS (MALDI, DCTB): 3180.6871 ([M+Na]$^+$); calculated for $C_{159}H_{252}N_6O_{57}Na$: 3180.6897.

Compound 2. To a solution of **1** (25mg, 7.9 µmol) in 2 mL of CH_2Cl_2, $Zn(OAc)_2\cdot 2H_2O$ (3.5 mg, 15.8 µmol) in 2 mL of MeOH was added. The mixture was allowed to react and, after 2 hours, the crude compound was concentrated under reduced pressure and purified by flash column chromatography (SiO_2, CH_2Cl_2/MeOH 25:1). The pure compound was obtained as a purple sticky oil without further purification (21 mg, 83%). IR (ATR): 3488 (br), 2872 (s), 1712 (m), 1664 (m). 1454 (m), 1434 (m), 1350 (m), 1106 (s), 942 (m); ^{1}H NMR (500 MHz, $CDCl_3$): 9.04 – 8.94 (m, 6H), 8.84 (m, 2H), 8.28 (d, ^3J = 8.0 Hz, 2H), 8.16 (d, ^3J = 8.0 Hz, 2H), 7.38 (d, J = 1.8 Hz, 6H), 7.18 – 7.09 (m, 1H), 6.90 (d, ^3J = 1.8 Hz, 3H), 6.86 (s, 1H), 4.26 – 4.15 (m, 12H), 4.03 – 3.97 (m, 2H), 3.91 – 3.85 (m, 2H), 3.70 – 3.50 (m, 96H), 3.47 – 3.38 (m, 24H), 3.33 – 3.24 (m, 24H), 3.21 – 3.11 (m, 24H), 3.06 – 2.99 (m, 18H), 2.54 – 2.46 (m, 6H). ^{13}C NMR (125 MHz, $CDCl_3$): 171.6, 168.4, 158.5, 150.3, 150.2, 150.2, 150.1, 149.9,

145.3, 135.0, 134.9, 133.5, 132.4, 132.3, 131.7, 125.5, 121.2, 119.7, 114.8, 101.4, 72.3, 71.9, 71.9, 71.8, 71.0, 70.9, 70.8, 70.5, 70.4, 69.9, 67.0, 59.2, 59.1, 59.0, 40.4, 38.1. HR-MS (MALDI, DCTB): 3242.5995 ($[M+Na]^+$); calculated for $C_{159}H_{250}N_6O_{57}ZnNa$: 3242.6032.

CTPR4-1 / CTPR4-2 conjugation. Prior to any conjugation, purified **CTPR4** at a protein concentration of about 100μM was freshly reduced with 1 mM 1,4-dithio-DL-treitol (DTT) during 20 minutes to ensure full reduction of the cysteine residues. DTT was removed by buffer exchange over a NAP-5 column (GE Healthcare Life Science) in PBS buffer. Protein fractions without DTT were collected at 4°C and 1mM TCEP was added to avoid the formation of disulfide bonds between cysteines during the conjugation reaction. The protein concentration was measured by UV-absorbance. 300μl of 50μM of freshly reduced protein was mixed with 40 equivalents of **1** or **2** (around 1.9 mg) giving a ratio of 1:5 cysteine:maleimide and mixed gently. After an hour, an extra of 20 equivalents of **1** or **2** was added to the mixture (around 1 mg) giving a final ratio 1:7.5 cysteine:maleimide. The reaction mixture was incubated during 3 hours shaking and protected from light. 15% SDS-PAGE electrophoresis gels were used to monitor the conjugation process. Samples of the **CTPR4** protein control and marker were prepared using Amresco EZ-vision loading buffer and the conjugates were mixed with SDS loading buffer. The gel prior staining was imaged using UV-light to monitor the fluorescence of the porphyrins. Then, the gels were stained with Coomassie Blue.

MALDI-TOF Mass spectrometry. The samples were analyzed using ABi 4800 MALDI TOF/TOF mass spectrometer. 4-Hydroxy-3-5-dimethoxycinnamic acid was used as matrix at 10mg/ml dissolved in a 70% acetonitrile and 0,1% TFA solution. The samples were prepared at a sample to matrix solution ratio of 1:4 (v/v) and 1µl of the mixture deposited on the sample plate. When the sample dried, 1µl extra of matrix was deposited over the sample.

Gel filtration chromatography. To purify the conjugate from the free porphyrins gel filtration chromatography was performed using an AKTA prime plus Fast Protein Liquid Chromatography (FPLC) equipment (GE Healthcare). The conjugation reaction was injected into a Superdex 75 HR 10/30 size exclusion chromatography column (GE Healthcare) and run at 0.5 mL/min in PBS buffer with 2mM β-mercaptoethanol. The purified samples were collected in 0.5 mL fractions and stored protected from light.

Absorbance measurements. Absorbance spectra were recorded using a VARIAN-80 UV-*vis* spectrophotometer. The absorbance spectra of the protein, porphyrin, and protein-porphyrin conjugates from 230 nm to 1000 nm were acquired in a 1 cm pathlength quartz cuvette using a 4 nm slit-width. For the denaturalization experiments, absorption spectra of the conjugates in different percentage of methanol were recorded. The equilibrium

denaturation studies were performed by preparing two stock solutions at 5µM of **CTPR4-1** and **CTPR4-2** in a PBS buffer and in methanol. The two solutions were mixed at different ratios to obtain solutions of conjugates at the same concentration and at different percentage of methanol (from 0 to 100%).

Film formation. Solid CTPR protein ordered films were generated as previously described.[15b] **CTPR4** protein alone and porphyrin conjugates **CTPR4-1** and **CTPR4-2** were diluted to 3% (w/v) protein concentration in 10 mM NaCl, 10 mM Na phosphate pH 7.0 buffer. The solutions were deposited on different surfaces, depending on the experiments to be performed. Quartz cuvette was used for CD analysis and fluorescence spectra, quartz plate for conductivity measurements, and silicon wafer for XRD analysis. The drop volumes also vary between 10 to 30 µl. The solvent evaporated at room temperature during 12 hours, resulting in solid thin films.

Circular dichroism (CD) measurements. CD spectra were measured using a Jasco J-815 CD Spectrometer. CD spectra of CTPR protein and **CTPR4-1** and **CTPR4-2** conjugates in PBS buffer were acquired in a 1 cm path length quartz cuvette at protein a concentration of 1.7 µM. CD spectra of **1** and **2** were acquired under the same conditions using 1.7 µM of porphyrin concentration. All the CD spectra were recorded with a band-width of 1 nm at 1 nm increments and 10 second average time. CD spectra of CTPR protein films were acquired in 0.01 cm pathlength quartz cuvette. 10 µl of the conjugate at 1% (w/v) protein concentration in 10 mM NaCl, 10mM Na phosphate pH 7.0 buffer were deposited on the quartz cuvette, and the solvent was left to evaporate. The CD spectra were recorded at 1 nm increments and 10 second average time. 10 scans were accumulated.

Fluorescence anisotropy measurements. Fluorescence anisotropy experiments were recorded in a Fluorolog–TCSPC spectrofluorometer (Horiba) with excitation and emission polarizers. Protein films were placed on a quartz sandwich cuvette and the fluorescence intensity signal of the film of the **CTPR4-1** and **CTPR4-2** conjugates was monitored. The samples were excited at 420 nm with a 4 nm slit-width and the fluorescence emission of the porphyrins was recorded at 650 nm with slit width of 4 nm. The fluorescence intensity was recorded with a fixed polarizer at 0 degrees in the excitation path and varying the angle of the polarizer placed in the emission path. The change of the intensity of the porphyrin in solution was recorded to account the difference in transmission efficiencies when the emission polarizer is placed at different angles. The intensity signal of the film was corrected for this change and then calculated the intensity change with respect to the fluorescence intensity (FI) when both polarizer are placed at 0 degrees (FI/FI_0).

X-ray Diffraction (XRD): X-ray diffraction was performed in a Panalytical X'Pert PRO diffractometer with Cu tube (lambda Kα=1.54187 Å) operated

at 45 kV, 40 mA, Ni beta filter, programmable divergence and anti-scatter slits working in fixed mode, and fast linear detector (X'Celerator) working in scanning mode.

FP-TRMC and TAS measurements. Charge carrier mobility was evaluated by flash-photolysis time-resolved microwave conductivity (FP-TRMC) and transient absorption spectroscopy (TAS) techniques at room temperature under air. Solid **CTPR4-1** and **CTPR4-2** films were prepared by drop-casting of their Milli-Q water solution. Charge carriers were photochemically generated using a third harmonic generation (λ = 355 nm) of a Spectra Physics model INDI-HG Nd:YAG laser with a pulse duration of 5–8 ns and frequency of 10 Hz. The photon density of a 355 nm pulse was 9.1×10^{15} photons cm^{-2} $pulse^{-1}$. The microwave frequency and power were set at ~9.1 GHz and 3 mW, respectively. The TRMC signal, picked up by a diode (rise time < 1 ns), was monitored by a Tektronics model TDS3032B digital oscilloscope. The observed conductivities were normalized, given by a photocarrier generation yield (ϕ) multiplied by sum of the charge carrier mobilities ($\Sigma\mu$), according to the equation, $\phi\Sigma\mu = (1/eAI_0F_{light})(\Delta P_r/P_r)$, where, e, A, I_0, F_{light}, P_r, and ΔP_r are unit charge of a single electron, sensitivity factor (S^{-1} cm), incident photon density of the excitation laser (photon cm^{-2}), correction (or filling) factor (cm^{-1}), and reflected microwave power and its change, respectively. TAS measurements were carried out at room temperature under air. The identical drop-cast films used for FP-TRMC measurements were used for TAS measurements. The film was photoexcited using a third harmonic generation (λ = 355 nm) of a Spectra Physics model INDI-HG Nd:YAG laser with a pulse duration of 5–8 ns and frequency of 10 Hz, where the photon density of a 355 nm pulse was 9.1×10^{15} photons cm^{-2} $pulse^{-1}$. A white light continuum from a Xe lamp was used as a probe light source for transient absorption spectroscopy. The monochromated probe light was guided into a Hamamatsu model C7700 wide-dynamic-range streak camera system, which collected a two-dimensional image of the spectral and temporal profiles of light intensity.

B. Supporting Figures.

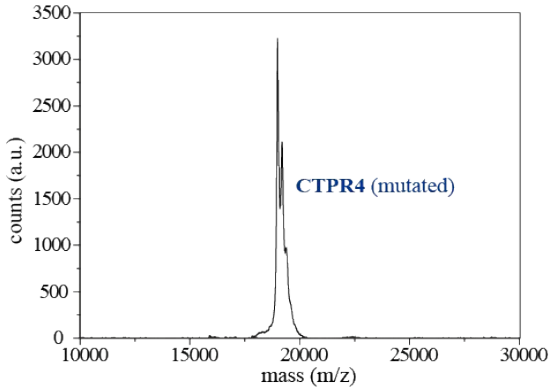

Figure S1. MALDI-TOF spectrum of the **CTPR4** protein mutated with eight cysteine residues.

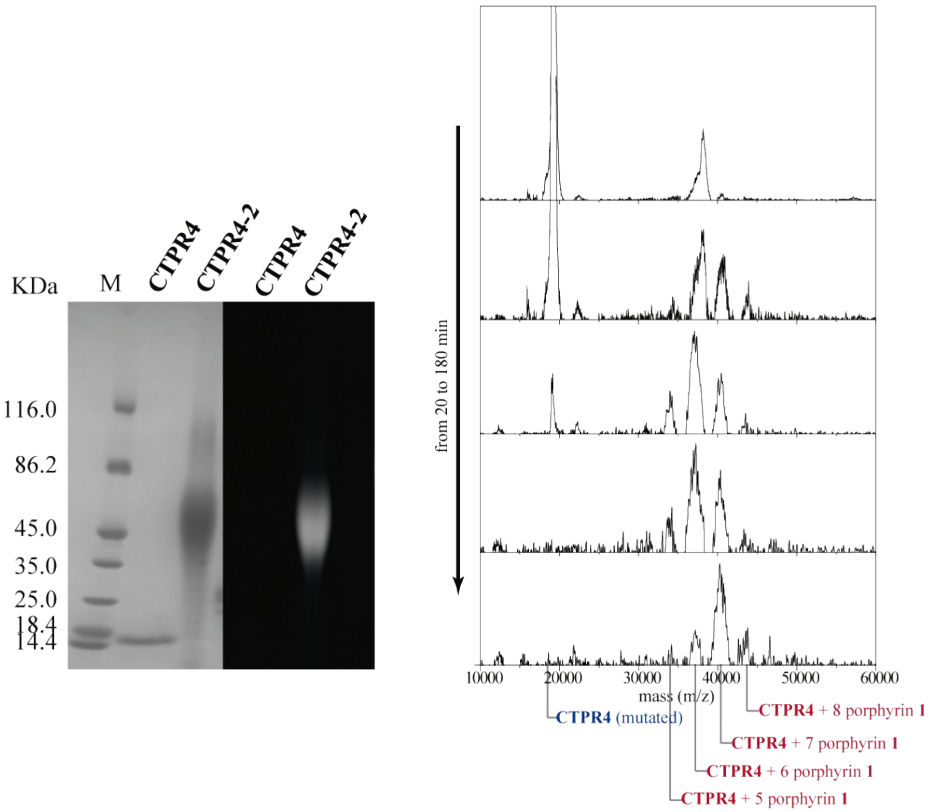

Figure S2. Left: SDS-Page electrophoresis gel of the **CTPR4-2** conjugate. The gel is imaged using Coomassie Blue staining (left panel) and UV-light to monitor the fluorescence of the porphyrins (right panel). Right: MALDI-TOF spectra of the conjugation reaction in **CTPR4-1** at different reaction times.

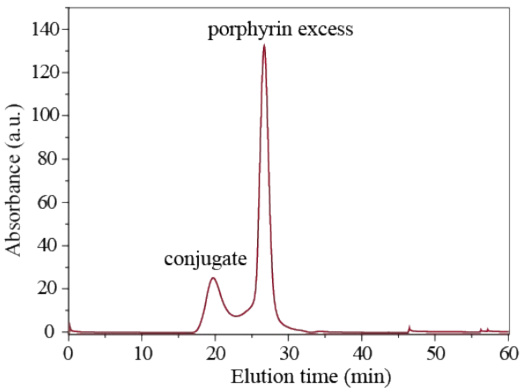

Figure S3. Size exclusion chromatogram of the purification process of the conjugate from the excess of porphyrin.

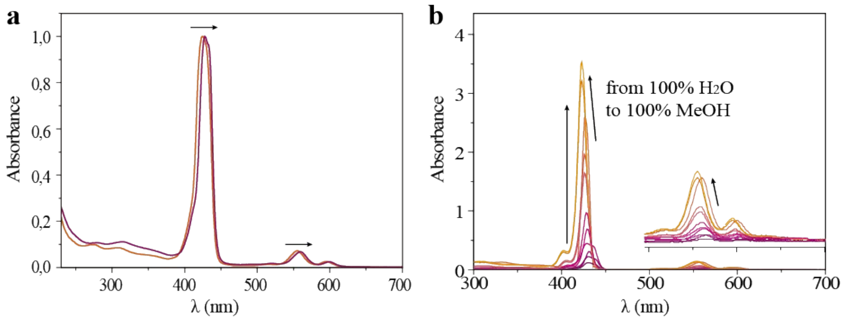

Figure S4. (a) Normalized UV-*vis* spectrum of **CTPR4-2** conjugate compared to normalized UV-*vis* spectrum of **2** in PBS buffer, showing the changes in the Soret and Q-bands. (b) UV-*vis* spectra of **CTPR4-2** (0.1 μM) at different methanol concentrations. Arrows show the increase of the methanol concentration from 0 to 100%.

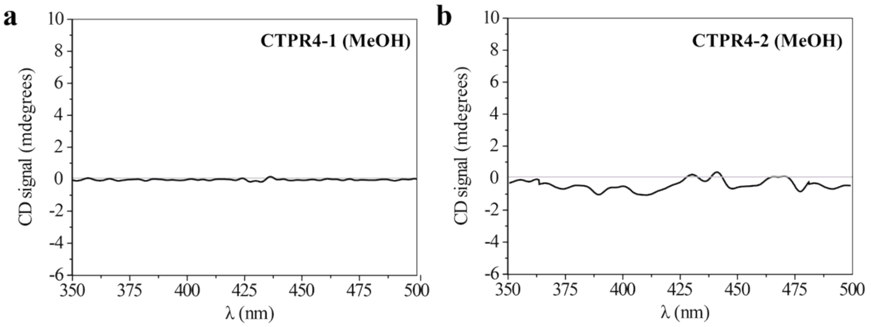

Figure S5. CD spectra of **CTPR4-1** (a) and **CTPR4-2** (b) in methanol.

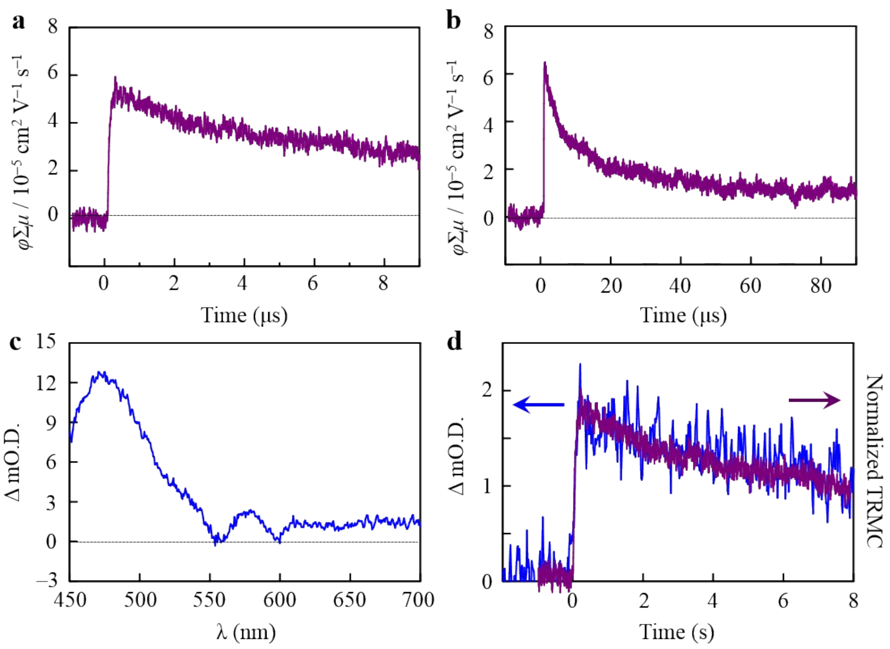

Figure S6. FP-TRMC kinetic traces of a film of **CTPR4-2** at (a) 0–8 μs and (b) 0–80 μs time ranges. (c) Snapshot of transient absorption spectra of a film of **CTPR4-2** at ca. 0.2 μs. (d) Kinetic traces of transient absorption spectra at 530 nm (blue) and normalized FP-TRMC profile (red) of a film of **CTPR4-2**.

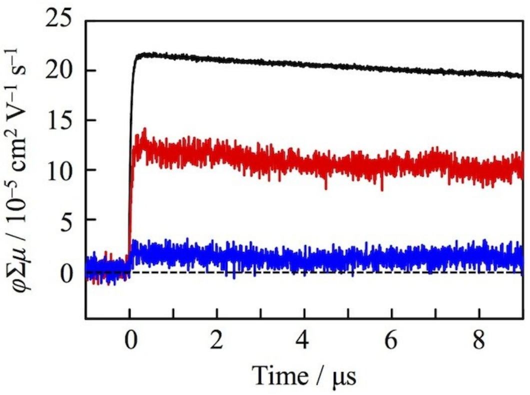

Figure S7. FP-TRMC kinetic traces of a film of **CTPR4** photoexcited at 266 nm (black), photoexcited at 355 nm after 266-nm irradiation (red), and photoexcited at 355 nm (blue). When the film was photo-excited at 355 nm after the measurement by 266 nm photoexcitation, a larger $(\varphi\Sigma\mu)_{max}$ value was observed which indicates the irreversible damage of the film. Overall, **CTPR4** itself gave charge carriers by irreversible photo-damages. However, when porphyrin chromophores are attached to the CTPR4 scaffold, porphyrins absorb light. The observed photoconductivity for **CTPR4-1** and **CTPR4-2** in the manuscript was due to the consequence of porphyrin arrays.

^{1}H NMR and ^{13}C NMR spectra for All New Organic Compounds

^{1}H NMR (500 MHz, $CDCl_3$) spectrum of **5**

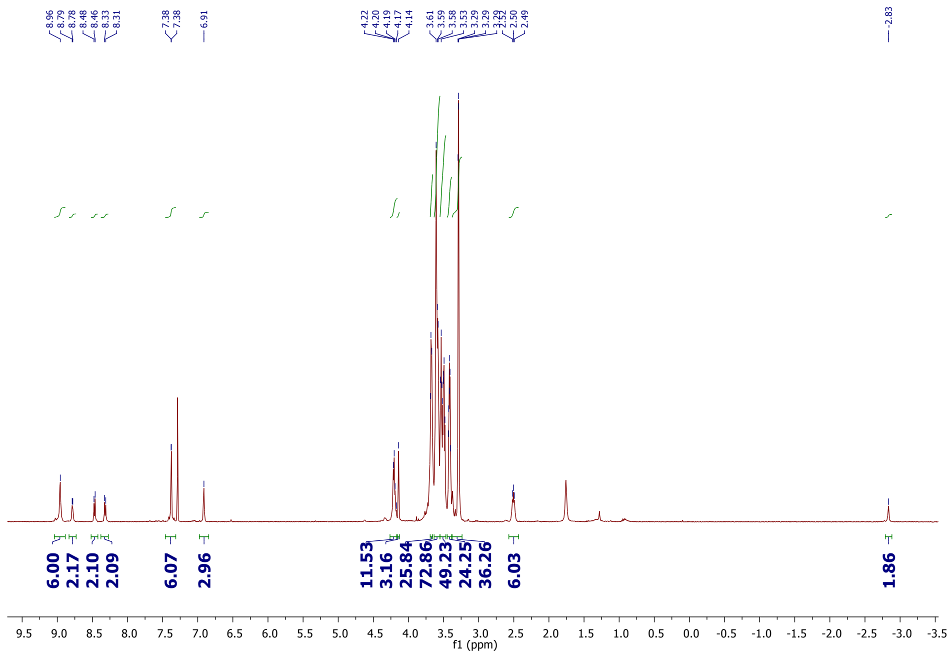

^{13}C NMR (125 MHz, $CDCl_3$) spectrum of **5**

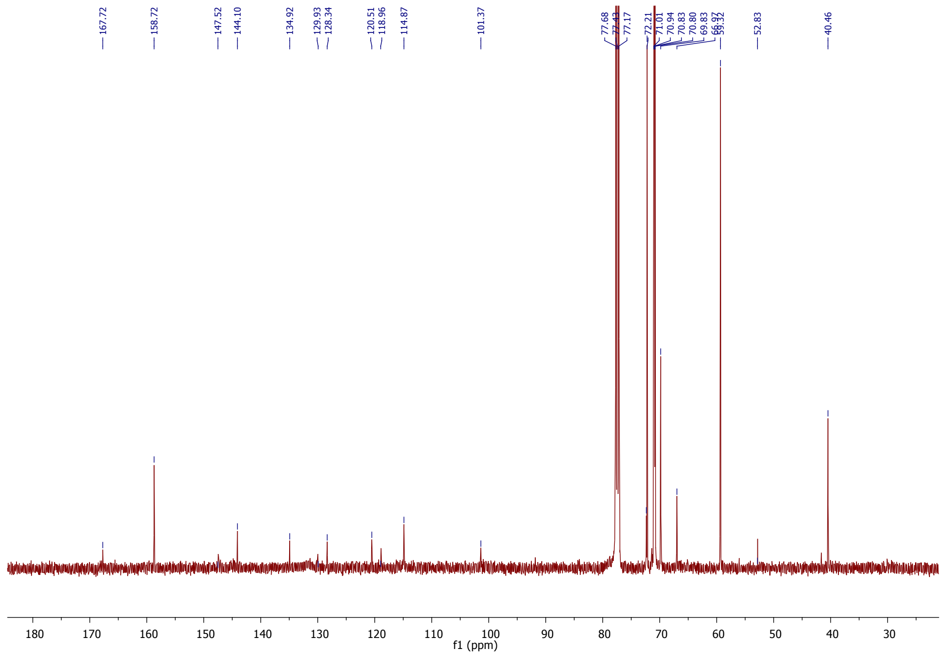

^{1}H NMR (700 MHz, $CDCl_3$) spectrum of **6**

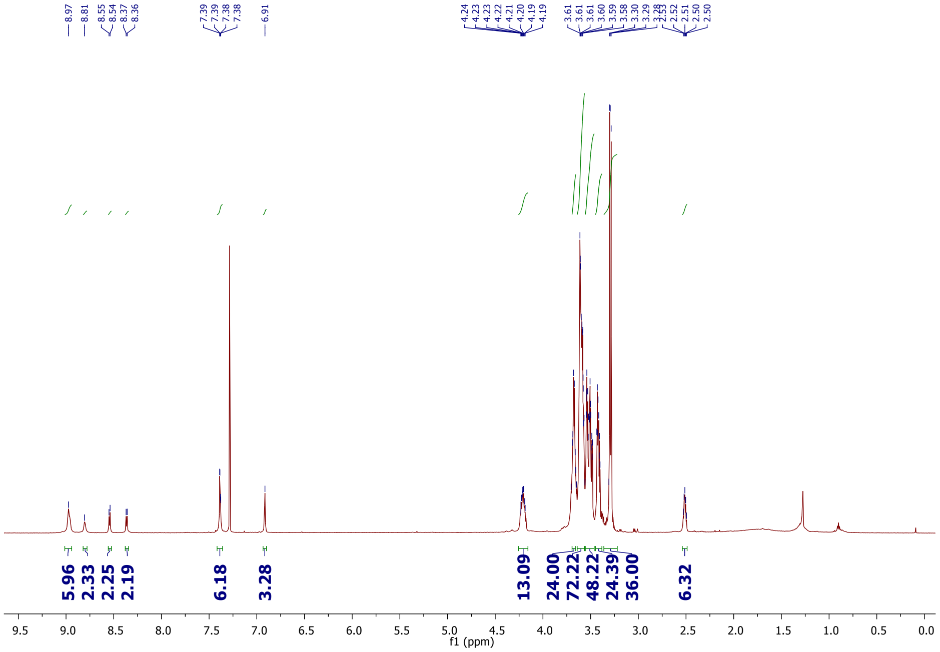

^{13}C NMR (175 MHz, $CDCl_3$) spectrum of **6**

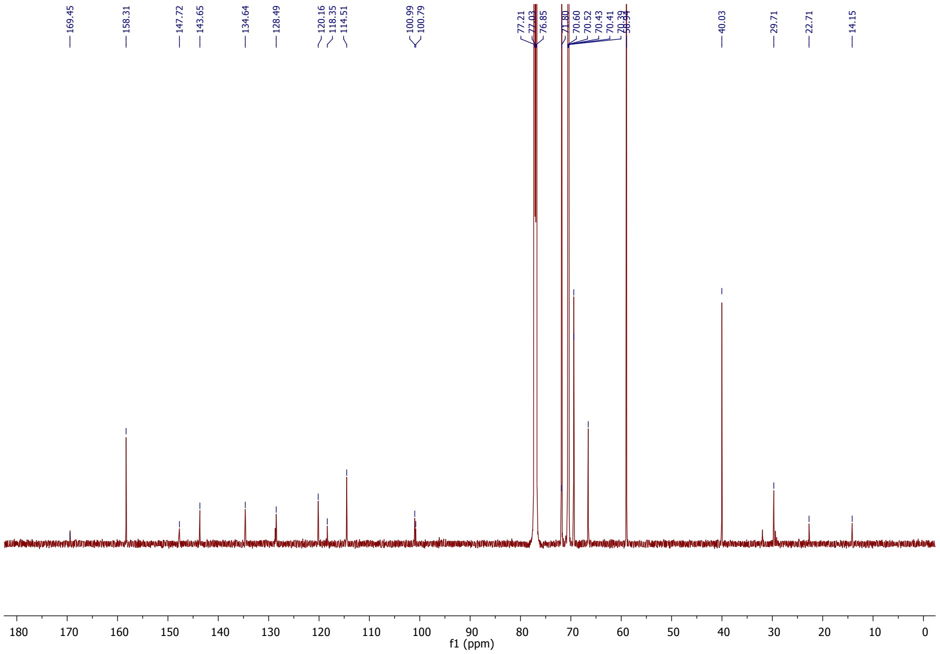

^{1}H NMR (500 MHz, $CDCl_3$) spectrum of **1**

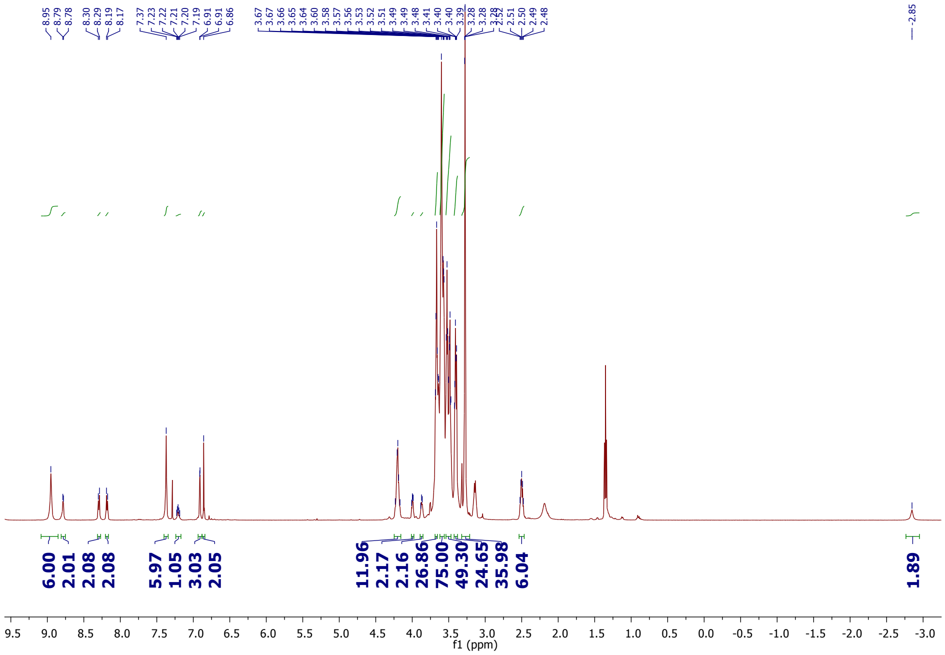

^{13}C NMR (125 MHz, $CDCl_3$) spectrum of **1**

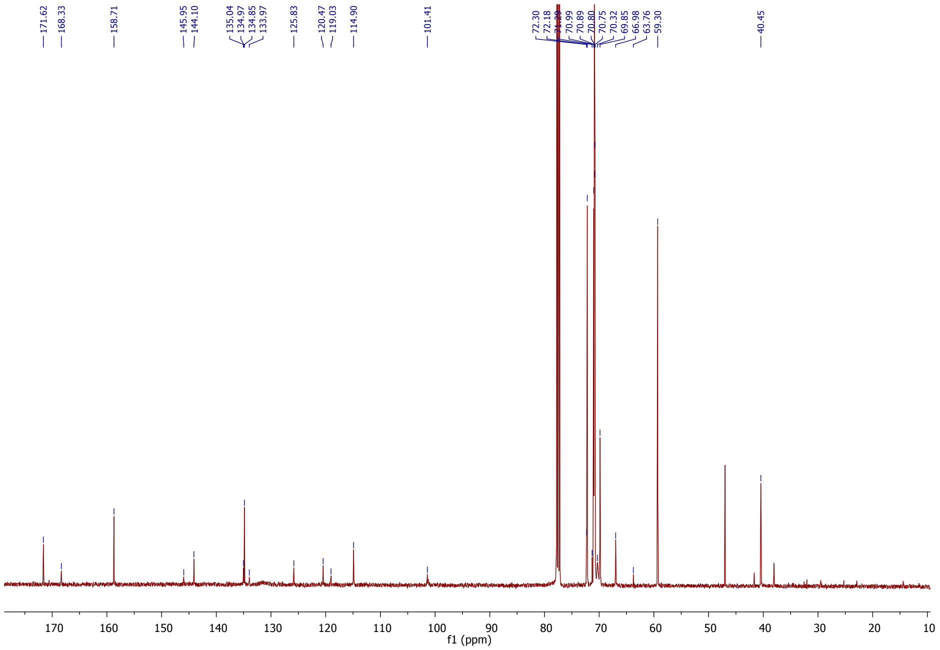

^{1}H NMR (500 MHz, $CDCl_3$) spectrum of **2**

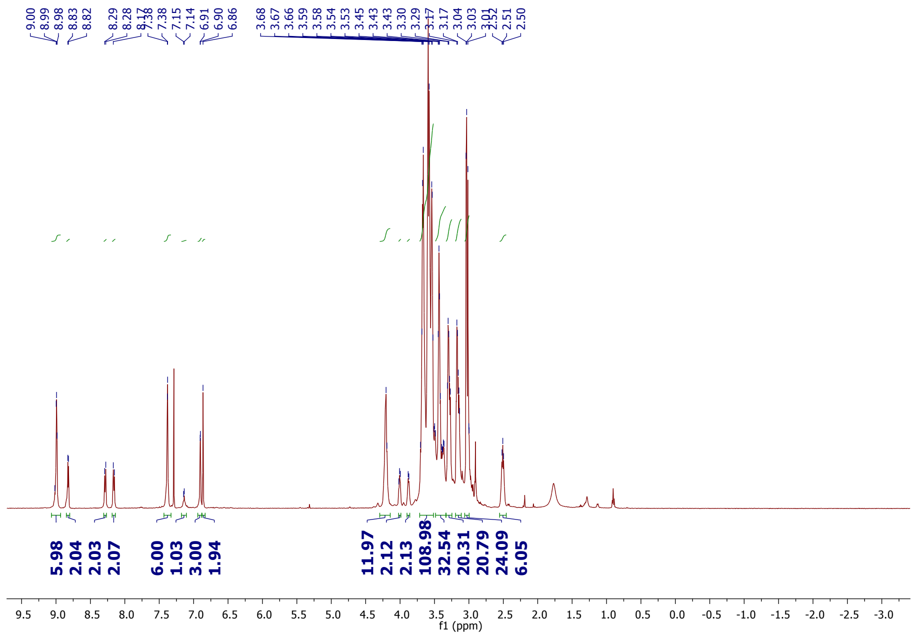

^{13}C NMR (125 MHz, $CDCl_3$) spectrum of **2**

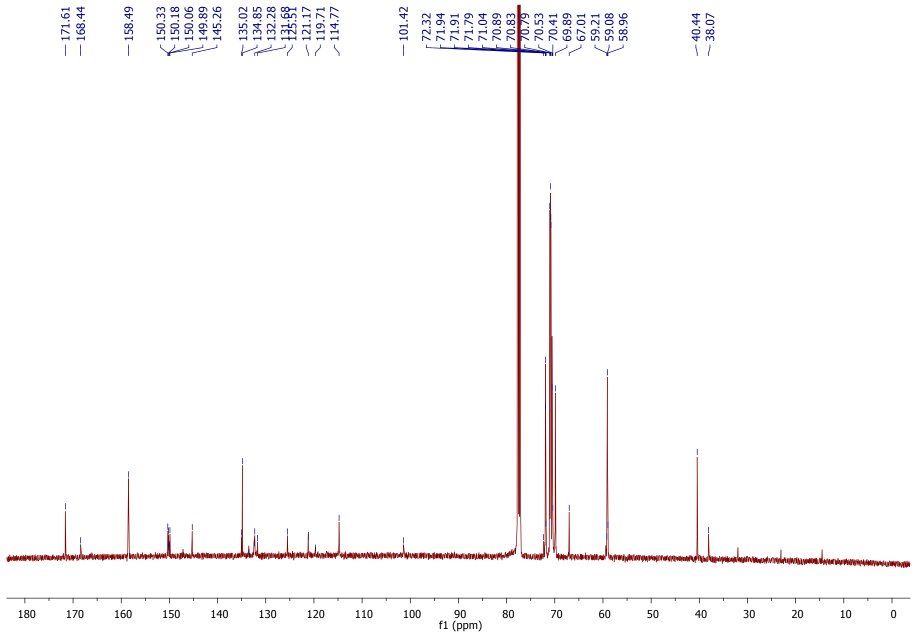

Supplement: Supplementary file 1 [file SC-007-C6SC01306F-s001.pdf]
